# Social determinants and chronic kidney disease of undetermined origin in childhood: Its communication and understanding described by families in Lake Chapala, Mexico

**DOI:** 10.3389/fneph.2022.962887

**Published:** 2022-10-26

**Authors:** Norma Guadalupe Ruiz-Velazco, Felipe de Jesús Lozano-Kasten, Horacio Guzman-Torres, Aaron Ismael Mejía-Sanchez

**Affiliations:** ^1^ Nursing Department for Care, Development and Community Health Preservation, Centro Universitario de Ciencias de la Salud, Universidad de Guadalajara, Guadalajara, Jalisco, Mexico; ^2^ Environmental Health Department, Centro Universitario de Ciencias de la Salud, Universidad de Guadalajara, Guadalajara, Jalisco, Mexico

**Keywords:** chronic kidney disease of undetermined origin, childhood, qualitative research, environmental determinants of health, social determinants of health

## Abstract

This work is based on the recognition of the existence of a complex relationship between social and environmental determinants and infants with chronic kidney disease of non-traditional etiology (CKDnT). The aim is to understand how the Social and Environmental Determinants are settled and its influence to the CKDnT in childhood, through knowledge built from the population that has lived the experience of this disease. This research was carried out with a narrative-conversational design. The experience of CKDnT was organized in stories focused on the experience of families in the social and environmental context where they live, get sick, suffer, and die from the disease. In the dialogue emerges the intersection of the social determinants of the disease, the different ways of life, and the relationship with the health services that attend them.

## Introduction

In the last 40 years, chronic kidney disease of non-traditional origin (CKDnT), has presented an increase in young inhabitants of agricultural and vulnerable communities located in several countries of Central America, who have a severe form of renal failure of unknown etiology. The Pan American Health Organization, for its acronym PAHO, has named it “chronic kidney disease of non-traditional causes” or CKDnT. This kidney pathology consists of a type of chronic interstitial nephritis that has reached epidemic proportions, causing high mortality in these communities and the overcrowding of their health systems: “According to a recent analysis, in Central America there were between 1997 and 2013 more than 60,000 deaths from renal failure (indirect indicator of CKDnT); 41% of them in people under 60 years of age (1). The highest mortality rates were recorded in countries such as El Salvador, Nicaragua, Belize, Costa Rica, Guatemala, Honduras, Panama and Mexico (2). Although the etiology of this epidemic has not been fully clarified, it has been observed that this disease is mostly located in farming communities with certain characteristics. For example, many affected communities and regions in Central America are located in low-altitude areas with high temperatures and close to the Pacific coast, since this geographical area contains the largest portion of arable land (1). Although now it is understood that CKDnT has its origin from multiple determinants of health, it is currently based on two main hypothetical mechanisms most likely interdependent: exposure to agrochemicals and agricultural work practices. The two mechanisms are directly related to precarious working conditions, the social vulnerability in which those communities live and the tropical climate (3).

## Social determinants of health and CKDnT

Kidney disease is a pathological process that arises from biological determinants, however, the environmental, social, cultural, gender, economic and environment factors play a determining role, since it is in these spheres, where health inequality begins.

In 2005, the World Health Organization defined the social determinants of health as: “the circumstances in which people are born, grow, live, work and age, including the health system” (4). Subsequently, in 2012, the Pan American Health Organization indicated that, in addition to making visible the effects caused by these determinants on health, it is necessary to make evident how public policies help or not to improve the living conditions of populations (5).

As the social determinants of health are distributed with greater inequality, health conditions for people are affected resulting in the increase in the prevalence of diseases such as Noncommunicable Diseases, in which factors such as income, educational degree, gender and type of employment, have a great influence on the care and control of diseases such as diabetes, hypertension and mortality (6).

The authors Cortés-Sanabria et al., point out that in a country facing demographic transitions, a precarious health system, an unstable political environment, lack of access to renal replacement therapies, more marginalized groups living immersed in conditions of social inequality and weak primary care, will result in a fairly complex management of CKD (7).

A literature review carried out by authors Hernandez Gamundi, et al. (8) concludes that the CKD affects the physical, psychological and emotional state of those who suffer from it, in addition to generating personal and family precariousness, where inaccessibility to social security is one of the determinants that mostly reduce their economic income. It is usual that only women are whom stands social support to their CKD family members, and this means facing all the negative circumstances associated with this disease. Therefore, it is of vital importance to understand and address the social determinants of health such as income, health system, culture and gender among others (8).

## The Agua Calientecommunity context

The Agua Caliente town is located at the Lake Chapala region in the municipality of Poncitlan in the state of Jalisco, Mexico. Lake Chapala is the largest nationwide and third in Latin America. It is located in the western part of Mexico in the states of Jalisco and Michoacán. Its area covers approximately 114,659 hectares, with 86% of these in Jalisco territory. Chapala Lake is part of the Lerma-Chapala basin which originates southwest of Toluca (9). Ten years ago, Chapala lake received the designation of wetland of international importance and of it belongs to the convention of places protected by the intergovernmental environmental treaty established by UNESCO “Treaty of Ramsar” (9).

The population in Agua Caliente have about a thousand people where children represent an important portion; in general, families are made up of mother and father and an average of 4 to 6 children. It is believed that their ancestors are originally from the Nahua tribes, although at present days, they do not preserve neither clothing nor indigenous dialectic (10).

This community lives on an exceptional circumstance of poverty, marginalization and structural social exclusion, which leads to increased biological susceptibility and reduced immune response, so, this is not favorable when diseases appear. However, we are talking about a community that lives a reality of socioeconomic limitations and on a degraded environment (11).

The Agua Caliente community inhabitants can reach to health services through a government-funded facility named “Health House”. This facility has two offices with an examination room, where regularly a doctor and a nurse attends in the morning twice a week (4 hours) giving medical assistance mainly to mothers and her child. There is common to have scarce or no availability of medicines or healing material, so, in order to reach these, patients have to travel 18 kilometers to the head of the municipality to buy medicines or have medical assistance when there is no doctor in their community. The absence of medical assistance in Agua Caliente is always on the afternoon, night and weekends). However, the demand for these health House services by inhabitants is scarce, due the vast majority go to private doctors in the municipal capital, with a higher costs of transportation (field notes, April 2019).

A group of patients and its relatives residing in of Jalisco state, Mexico, agree that getting access to hemodialysis treatment has drawn several economic, food, family and transportation problems. All of the above are associated with lack of economic solvency to sustain the costs related with disease, in addition to having precarious jobs and no social security (12).

In Agua Caliente, the prevalence of malnutrition in preschoolers was 39.3% in its three categories while in schoolchildren it was 34.1% which were considered very high compared to the national prevalence of 3.9% according to the National Survey of Health and Nutrition of Medio Camino ENSANUT MC 2016 (13).

## The life in Agua Caliente

The average schooling in men is primary completed, in the case of women it is of truncated primary (Field notes, April 2019). In common terms, people are dedicated to the cultivation of beans, corn and fishing for personal consumption, while the chayote is mainly cultivated for marketing. About the men, the masonry trade is common and in women the work as a domestic worker in Guadalajara, although, in general, men are responsible for supporting the home economically while their partners take care of domestic chores. The average weekly income per family is approximately 900 to 1200 Mexican pesos (Field Notes, April 2019).

People in Agua Caliente suffer from the lack of clean drinking water, as they only have it two or three times a week and only 3 to 4 hours a day. In order to solve this problem, they make use of the water of Lake Chapala, either for bathing, washing clothes, kitchen dishes, and anything needed to be cleaned. Many of the houses have no toilets and use latrines, the floors of the houses are mostly dirt and it is usual that three to four people sleeps for each room (Field Notes, April 2019).

The use of agrochemicals is common among those who are engaged in agriculture. These are acquired without any age restriction in the municipality downtown of Poncitlan. It is easy to buy agrochemicals due to is not necessary to know the name of the product to acquire it, or having any kind of license for it, so, it is enough to ask for the color and know what it is used for. The problem with this practice is the abuse of the quantities and the appropriate handle for its use, resulting in a higher risk of acute or chronic poisoning (Field Notes, April 2019).

## Chronic kidney disease of non-traditional origin in Lake Chapala

Epidemic CKD of unknown or undetermined cause has a geographical, social, economic, and political environment. It exists in a delimited territory, and as such, has specific natural characteristics in common, however it has allowed, for centuries, a harmony between the good life of these communities, in the territory of Lake Chapala. They are located on the northern shore of Lake Chapala at 1,500 meters above sea level, in a strip of land 10 kilometers long by 100 to 1000 meters. This territory is home to approximately 15,118 inhabitants, distributed in the localities of Mezcala 6,000 (40%), San Pedro Itzicán 7,500 (50%), Agua Caliente 1050 (7%), El Chalpicote 568 (3%). There are significant delays and gaps in health, education, food and nutrition, effective access to drinking water, employment, which have a negative impact on the population and especially on children and young people, a situation that is linked to an epidemiological profile in this territory characterized by the overlapping of infectious diseases and non-communicable diseases such as malnutrition in schools, kidney disease, related to living conditions and agricultural work and fishing. Among the non-communicable diseases of chronic tendency are persistent albuminuria, chronic renal insufficiency, malnutrition, obesity, accidents, cancer, alcoholism, drug addiction, endocrine disorders, and more that are related to the type of food. In the political environment, the health policies deployed in the region are oriented to maintain little for all, with a minimum and controlled level of needs and limited accessibility. In the area of Human Rights, there is resolution 7/2020 of the Inter-American Commission on Human Rights (IACHR) dated February 6, 2020, which considers that the complaint meets *prima facie* the requirements of gravity, urgency and irreparability contained in Article 25 of its rules of procedure. Consequently, the Commission requests Mexico to: 1.- Adopt the necessary measures to protect the lives, personal integrity, and health of the inhabitants of the area up to 5 kilometers from the Santiago River in the municipalities of Juanacatlan and El Salto, as well as the inhabitants of the towns of *San Pedro Itzicán, Agua Caliente, Chalpicote, and Mezcala in the municipality of Poncitlán*, State of Jalisco, indicated in the petition, and in particular, 2, that the State adopt the pertinent measures to provide a specialized medical diagnosis for the beneficiaries, taking into account the alleged contamination, also providing them with adequate medical care in conditions of availability, accessibility, acceptability and quality, in accordance with international standards, in this complex environment, CKD of unknown etiology was mainly observed by the population and the health services in the early 1990s. More than 30 years have passed, and the public perception is that CKD has increased. Some of the population and human rights defenders claim that the cause is to be found in the thermal water that is delivered to the communities from deep wells in the area or in the water that is extracted from Lake Chapala, although few inhabitants drink it. To date, there are no studies or data that could explain the high prevalence and/or causes of decreased glomerular filtration rate (GFR) in this population. Previous water quality studies carried out in this region by different governmental and social organizations determined heavy metals, and their results are within the Mexican normative regulations. In these localities, the prevalence of albuminuria in school children is 3 to 5 times higher than that reported in the international literature (14).

In a prospective cohort study conducted in the rural community of Agua Caliente, no clear cause of CKD was identified. More studies are needed to detect the risk factor(s) involved, where 15 out of 1000 schoolchildren have CKD, and the prevalence of albuminuria is 47.5% (15).

## Materials and methods

In order to access the experiences of people suffering from non-traditional chronic kidney disease, we carried out a qualitative, phenomenological-descriptive study based on Schütz’s social phenomenology. The descriptive phenomenology reflects the interest and need to capture, describe and understand the intentionality of the investigated subject and the meaning of his experience in the concrete situation in which he finds himself (16).

The sampling carried out was purposive (17). The size of the sample to achieve saturation of information (18, 19)was made up of ten informants from different families. For the collection of information, participant observation was used (20) in-depth interview (20, 21), and informal dialogues. The analysis was carried out from the proposal of the seven steps of Colaizzi (22).

## Results

The study shows the social determinants from the analysis of the distinctions made by people with CKD in this community. Grouped under the following subheadings:

### Cultural determinants

According to Kleinman, Eisenberg, and Good, the way we cope with, perceive, and experience illness and disease is based on the position we occupy in the social system in which we live (23). Thus, we all learn “approved” ways of being sick, of caring, of accompanying, and so on. It is not surprising, then, that there can be marked interculturality and historical variations in how chronic illness is manifested and treated over time.

For example, in Agua Caliente, kidney disease prefers to be treated in silence; people do not usually communicate that their sons or daughters are sick because it implies being stigmatized by the rest of the population. The appearance of the sick body is one of the main causes of mockery and rejection; in addition, the false idea that the disease can be contagious increases social exclusion. Johana narrates an episode where her grandson was the target of mockery and rejection. She remembers the experience as difficult and sad. In spite of trying to ignore those who were violent towards her grandson, it was painful for her to see him being excluded because he was ill:

*Johana: I just pray and tell God: “No more, God, don’t send us this illness. It is very hard because many people sometimes make fun of us because we were so sick, and I tell them: “don’t make fun, the world is very big and the world turns around. We don’t know.*

*Interviewer: Hey, but tell me, how do you make fun of them?*

*Johana: Well, many children would say, well, the little boy would say to me: “Mom, I don’t go out anymore” and I would say to him: “Why? What’s wrong with you? “And he would say to me: “Ama, I’m not going out anymore because the kids tell me that I’m fat, that I’m fat because of my kidney.” I felt really bad and I would just tell him “let them let their mouth be made into a crackling, don’t listen to them” but he would get really sad and he would say to me “no, mom, but it hurts me that they say that to me”. He didn’t want to go out because they told him he was sick and that he was going to infect them, and it hurt me to be called that. He felt sad and he would touch his belly and say to me: “Mom, am I fat?” and I would say: “No son, you are not fat” but he knew and he would say to me: “then why do they tell me that? Sometimes they would yell things at him, well, not when he was older because he would kick them.*

It is needed to clarify this kid has a kidney condition that caused edema in his stomach. So, the belly inflammation is not related with obesity.

### Economic determinants

Getting sick with kidney disease also means impoverishment, especially in an area so economically vulnerable and without access to social security to support them. The cost of dialysis, medications, transportation to health centers and ongoing medical studies are the priority to which the family’s economic resources are allocated. On different occasions Martha has had to decide between buying what is necessary for her daughter’s treatment or buying food, gas or shoes for the rest of her daughters and sons. “We have had a hard time,” she says, alluding to the economic difficulties she is going through:

*God will tell how far we will go. Thank God we have done well so far with the medications: we bought this, we bought that, and well, we have had a hard time. We stopped eating something to buy what she really needs: syringes, medicine for her, that her blood pressure medicine is running out, that her vitamins are running out. We stop buying something that is not necessary, that this is more necessary, well, this is good. My children, well there are times when they tell me “I need shoes” and I tell them “well, me too, but what do we do”, “we already know” they tell me. Like the older one, for example, goes to high school, the other child also goes to high school, and I say “Oh no, so much money for you, so much for you” and I think “and tomorrow what are we going to do? There are days that we have, there are days that we don’t, but we have gone out, well, here we are, we are still here. We’ve had a stove for a while now, and we have a gas tank that we haven’t been able to fill (laughs), and we say “Well, it’s better with firewood”, we cook with firewood. Sometimes the kids go to school early and what do we eat with? Well, there’s no gas to make them something to eat, no way! We’ve been wanting to buy gas for a while now and we want to buy gas and we just haven’t had the day, but yes, yes we have … in spite of everything and everything we have managed, as I said, to get ahead.*

For these people, due to the economic circumstances and social exclusion in which they live, kidney disease automatically means the arrival of death. For Luciana, accompanying her grandson to the hospital meant a complicated experience, as she understood beforehand that their economic situation was not in their favor and that the end result would be death:

*This kidney disease is a very bad illness, but it is worse when you are poor. My grandson at times was fine and at times he was bad and sometimes he went to the doctor and they gave him a painkiller or gave him an injection and they took his blood pressure…. Well, they gave him medicine, but it was an ordeal because I already knew that this illness cannot be cured, and even less so without money for everything they need. Here people die of it, and that’s how it was until he died.*

### Environmental determinants

People recognize the environmental configuration of their community and narrate how it has undergone severe changes through time and anthropic intervention. In these communities the interaction with the water of Lake Chapala becomes indispensable, since it is from the lake that the inhabitants build their daily life. Berenice, for example, remembers her father telling her that Chapala was prosperous, clean, harmless; it was not only a source of work, but also of life and recreation. She also assures that she drank water from there and it never harmed her.

*My dad used to take fish out of the lagoon to eat, he says there were lots of fish and everything: fish, frogs, crabs and lots of life. He and my uncles would swim and play. The water from the lagoon was drunk years ago, when the water was good and crystal clear; I also drank from there. By the time they were born (he points to his children’s nursery) the water was no longer drinkable.*

### Gender determinants

Being born male or female in these populations will condition the development of specific roles among the inhabitants of these communities. It should be remembered that roles are not assigned randomly but are attributed based on the cultural understanding of who are the ideal people to develop them. These assignments are known to be “natural” and, therefore, are unquestioned, taken for granted.

In the daily life of Agua Caliente and San Pedro, the role of primary caregiver is automatically assigned to the women of the household, generally, to the mothers or grandmothers of the renal patient. Thus, these women will be responsible for the care required by such member, besides being responsible for the rest of the domestic activities and care of the other children, since these tasks are in no way relegated to someone else in the family. This results in the multiplication of tasks that fall on a single family member: the primary caregiver. In Marina’s family, for example, she and her mother are responsible for food, laundry, and care and companionship for her brother. Her father, she told me, is not as familiar with his son’s care, as it is usually done by women:

*Marina: My mom and sometimes I, are the ones who feed my brother and, well, everyone in the house. We do the laundry, clean and, well, the chores in general. When my brother gets sick, my mom takes care of him or sometimes my sister comes here and helps.*

*Interviewer: What about your dad?*

*Marina: No, my dad doesn’t really know how to take care of him, he doesn’t know much about illness and things like that, I mean, he knows when he gets sick, but he, since he hardly accompanies him, well, he doesn’t. He doesn’t. It’s just that he’s already old and, well, you see, that’s more like women, one, well!*

The role assigned to men before and during the illness is that of provider. Men, as Marina describes above, are not very involved in their family member’s care, they do not know the treatments or their names, they do not usually go to the hospital, and they do not identify the doctors treating their loved ones. Their task is different from all of the above, they must be sure to provide the money needed to pay for the expenses incurred throughout the course of treatment. This agreement is never spoken about, but “naturally” family members take it for granted and generally accept it without protest.

*Martha: I’m always the one who stays in the hospital, because he works, because he works, because he has to work to help us get by, to have enough to eat there too because everything is very expensive there (referring to the prices of food in Guadalajara) and, anyway, he doesn’t know who the girl’s doctor is. He already goes on Saturdays and Sundays and accompanies me to the hospital for a while and then he comes back because he is going to work.*

In the common sense shared in the communities, caregiving is not considered as work, but rather as part of the natural obligations of being a woman/mother/grandmother or close relative of the sick person. In addition, the person who has an activity that is considered work (generally a paid activity) has sufficient justification for not taking care of the sick person, regardless of his or her relationship to the sick family member:

*Johana: My husband and my boy’s mother both worked, so I couldn’t take care of him because how could they stop working, and I took care of him when he felt sick.*

*Interviewer: You didn’t work?*

*Johana: No, I don’t work. I was just here at home doing chores and that’s why I took care of him because, as I said, I didn’t work.*

### Access to health care

In order to access health services in these populations, it is necessary to overcome a series of economic, cultural, structural and other obstacles. Most of the time, people prefer to resort to shared and inherited cultural knowledge, that is, to empirical remedies and care. This type of care, according to Menéndez (24)is constructed on the basis of collective knowledge that depends on the sociocultural circumstances in which it occurs and is renegotiated on a daily basis.

Johana exemplifies this well when she realizes that her grandson is “amalgamated” without knowing why and decides to opt for a home remedy that she used when she had blisters in her mouth and that her mother taught her was the right one. At first, the idea of going to the doctor was not an option until her grandson got worse. Lack of medical care and lack of resources to travel to a health facility is one of the main factors in people’s decision to opt for the common means of everyday life:

*We didn’t know what was wrong with my child, he just blistered no more than once suddenly. One day he said to me “look, mom, I got blisters in my mouth, and I can’t eat” and I told him “well, go wash your mouth out with carbonate” and that’s it. That time we didn’t take him to the doctor because, well, where here where so fast?*

In addition, people take advantage of the resources they have in their daily lives and previous knowledge to be able to care for their family members when they feel sick. Fernanda, for example, often uses herbal medicine and physical aids for headaches. Although she does not identify the relationship between cold/heat and physiological reactions, she knows empirically and from the knowledge inherited from her mother that her remedies are effective:

*With my other child … mmmm…. he started with a headache, he went to school and he told me that his head hurt and when we were washing he told me “I came to take a bath, mistress” and I told him “take a bath, the lagoon is very nice” and he jumped into the water and after a while he told me that his head didn’t hurt any more, He told me “back in school I felt like my head was breaking in half but with the bath it went away” and the pain did go away with the cold water, who knows why, but it works! But it would come out and it would hurt again and then I would make her a cup of tea or something to relax her, one of those things my mom used to make me and I saw that it did help!*

When this empirical knowledge is overwhelmed by the symptoms of kidney disease, the population chooses to seek medical help. The problem arises when the diagnosis comes too late, or misdiagnoses arise that generate confusion among families and delay timely care. For Martha, getting her daughter’s diagnosis was especially difficult; they had to visit several doctors, including specialists in pediatrics and nephrology, who assured that Julia had nothing more than a throat infection despite presenting more symptoms that were not typical of a respiratory tract infection:

*My little girl started with little purple welts on her legs, but we used to say “it’s mosquitoes that bite her” but no, because the mosquito feels the little ball and she had them under her skin and she scratched them and they came out like water, like this … thing. She was cured, they told me “Send her for tests” we sent her for tests and the doctor told me “No, she has nothing, she’s fine!” But she was swelling up, then he gave me a pass to the pediatrician in La Barca^1^. I went to the pediatrician and he said “No, it’s nothing, it’s probably a throat infection” but her feet were already swollen and so, well, we went and when we went for the second time he said “Bring her some new studies; get a renal echo and bring them all together”… and as I said, we had never experienced an illness in this family, I mean, I said “What should I do? What should I do? What should I do? Who should I ask? It was Thursday for a month and then I went to the studies in the morning that same day and I gave them to the doctor. The doctor saw them and said, “You know what, ma’am? the girl already needs to be hospitalized” I got scared and I said “Doctor, why? I didn’t know anything! You told me days ago that she didn’t have anything” And I went and they did the whole approach of everything, even the nephrologist said that it wasn’t the kidney and that nothing came out in the studies and I don’t know how much and she said that she didn’t have anything, but day by day she was swelling up, her hair was falling out a lot, she had a long braid, but it was falling out.*

Luciana says that sometimes the care patients receive is so inopportune that *“by the time you take your sick patients, well, thank you,”* referring to the fact that it is too late for them to receive effective treatment. She recognizes that the causes of the late treatment are poor medical diagnosis, lack of resources for timely studies and lack of knowledge about the disease:

*I saw my boy as well as very … very yellowish, very yellowish, by the way we took him to a doctor here in Ponci, he gave him medicine and he told us, “maybe he’s, ummmm, he’s lacking vitamins” but no, we never thought that they would do studies and everything, to know exactly the illness. And sometimes you don’t have money, you trust what the doctor says and then you don’t know and then you stay there and finally when you take your seriously ill patients, well, thank you! you can’t do that anymore.*

Moving to the hospital is one of the daily problems that are most complex to solve, because there are multiple economic, structural, and personal obstacles that require more than just the individual or family will to face them and be able to comply with the treatment of their loved ones.

One of the most frequent obstacles to receiving treatment is the inaccessibility of health centers for these populations, since in order to receive second or third level care alone^1^ it is necessary to travel at least 30 minutes by car to get to Poncitlán. However, most of the inhabitants of the communities do not have their own car and it is necessary to take a bus to get to the municipal capital and a bus to the Guadalajara central office:

*Interviewer: And what is it like to go to Guadalajara? How do you go?*

*Don Juan: In the car and then the bus from here to Ponci to there (to Guadalajara).*

*Interviewer: How far do you go there?*

*Don Juan: Well, first from here to Ponci - Poncitlán - and then another hour and a half to Guadalajara: two hours to get to the hospital, two hours.*

*Interviewer: And what time are the appointments?*

*Don Juan: In the morning, early, my son leaves here around five or five-thirty in the afternoon.*

In the case of San Pedro, the municipal government has assigned a special transport to take people to their dialysis treatments to Guadalajara. This transportation has an established schedule, in which it leaves the town Monday through Friday mornings and returns between three and four in the afternoon. In case of medical emergencies or laboratory studies at a different time, people must find a way to arrange for transportation. For example, Martha can’t always wait for the bus assigned to them in San Pedro because, as she tells me, when it is an emergency they can’t wait until the bus leaves and they resort to taking a taxi; the same thing happens when it’s time for tests and their schedules don’t coincide with the bus departure time and, if Julia feels well, they can walk from the old bus station to the old civil hospital. She rarely returns on the free transportation bus assigned for the community because her daughter gets desperate waiting for the bus to return with all the patients and their families:

*Martha: When we take the bus, we take the one that says Chapala at the bus station. When she’s doing well, when she doesn’t have anything, she walks to the hospital.*

*Interviewer: From headquarters to the hospital?*

*Martha: Yes, hold on (nods her head). When she gets sick, we must take a taxi; we have to take a taxi to take her to me so it will be faster. When we are about to leave here, I say to her “daughter, are you feeling well?” and she says “Yes, my love” and I ask her again “are you still walking? In other words, we don’t leave well, we walk slowly, and I ask her “are you tired yet?” and she says “noooo”, in other words, she feels fine and we get there fine and when we go to the appointment, they take tests, they take this or that, this normal one. There is also a transport that takes renal patients from here, but sometimes it doesn’t leave on time for our exams, or it doesn’t leave until four or five in the afternoon and we leave at eleven in the morning, and she is very desperate “mom, I want to go, I want to go, I want to go, I want to go to sleep” she says to me. And how I tell her, there are times when we leave at our own pace, but as long as we keep all our appointments, we come and eat and we are calm*.

Things are less favorable for the inhabitants of Agua Caliente, as they do not have official public transportation, thus forcing them to pay a private car to one of their neighbors who does have public transportation or to request a taxi. These services vary in cost depending on the time they are requested: a trip from the town to the municipal seat costs around 100 to 300 pesos. To go to Guadalajara, which is where they normally receive hospital care, varies from 500 to 1200 pesos, not including the return trip:

*Veronica: The difficult thing here is to get a truck to take him to the old civilian hospital, and you can see that it is far away. Sometimes you just need God’s help, because you see, sometimes there is no truck, there is no gas, they are out of order, well, you get desperate to see your sick person like this and there is no one to take him to the hospital.*

*Interviewer: And, do you have a car, or how did you manage to transport your child?*

*Veronica: No, we don’t have any. We get vans over there.*

*Interviewer: Who do you get it from, or who do you talk to?*

*Veronica: Well, sometimes here, with a man across the street, but when he has gas he does us a favor. In the early mornings … not in the early mornings, because not at night, but a cousin of mine sometimes takes us there too, he charges us two thousand pesos, sometimes a thousand pesos, even the civilian, according to him. And then the taxis from here to Ponci want to charge you a hundred, two hundred, three hundred, and it’s annoying.*

Sometimes the family members interviewed mentioned that it is more economical for them to stay in the shelter, in the hospital or even on the benches in the garden outside the hospital, since the main thing for them is that their loved one receives medical attention. Their own wellbeing or comfort *“however” they can* manage later:

*Fernanda: The important thing here is, well, that they take care of my boy, even though sometimes my husband had to sleep downstairs, on the floor, wherever, wherever, he slept downstairs. I would also go downstairs, I would sit over there, on a seat too, I would sit on the floor, wherever I could. Sometimes we got cardboard, not always, when we could. Then we started sleeping in a hostel, one nearby, we had been there for a month. Sometimes we couldn’t fit the two of us - there wasn’t enough room in the shelter for her husband and her - sometimes one night he and I, but it was okay because, for example, there were many people who couldn’t get through because there were so many of us and neither in one place nor the other - neither in the hospital nor in the shelter - and there they were on the park benches and, well, they were comfortable in the warmth, but in the cold? There you go around suffering, but you have your money counted, but well, whatever! but they take care of the sick … Well, that lasted almost like … more than six years, huh?*

## Discussion

For some time, there have been several approaches to the analysis of the relationship between socioeconomic inequalities and health-disease processes, mainly motivated by various academic and institutional actors. From the academic point of view, there are Latin American perspectives such as Social Medicine and Collective Health, which propose to look for the causes of inequality and its solution ways in what social determination means, authors such as Vicente Navarro, for whom it is not the inequalities that make people sick and kill, but those responsible for those inequalities, call the attention on the power relations that are hidden and on the responsible and beneficiaries of inequality (25).

Currently, there are several models regarding determinants, they explain the links between different types of social determinants and health, to allow to identify strategic points where policies should act; Dahlgren and Whitehead: Layered influences, Mackenbach et al: Selection and the causality relationship, Diderichsen et al: Social stratification and the production disease, Mackenbach et al: Selection and the causality relationship, The holistic model of Laframboise-Lalonde, Wilkinson and Marmot and their contribution from public policies, the model of the WHO Commission on Social Determinants. Therefore, it can be assumed that each country or region has particular characteristics, depending on the social system in which it lives, the prevailing ideology and the dominant culture (26).

The issues described along this manuscript as context of CKDnT, also have been described by authors where they pointed out in the fact of living in extremely poor conditions, as poor nutrition, lack of accessibility to health and diagnosis error. We believe this research contributes to understand the new forms of kidney disease of unknown etiology, that have affected populations in developing countries, these results raise the need for the development of social determinants schematized from a transdisciplinary approach, as a proposal to draw people knowledge and how they experienced this disease. This can be understood as a paradigm shift, and contribute to build holistic strategies on public health and to improve results in kidney health care (27).

## Key findings of the study

The reality of the Chapala riverbank cannot and should not be equated to the determinants that arise in a variable environment that has different characteristics such as culture, economy, ways of relating, communicating, social interactions, etc. An example of the non-uniformity of environments is reflected in the following findings.

- Women act as primary caregivers and are the ones who provide the main support for the renal patient: general care, feeding, accompaniment, treatment schedules are some of the activities entrusted exclusively to women. Men have the function of economic providers and do not provide any other type of care.
- Patient-doctor communication is often confusing, with people arguing that it is difficult to understand the language used by health care staff, which causes distress and confusion.
- The early diagnosis of renal disease, when the research began, was not available in this region due to the lack of economic resources, the lack of nephrologists close to the communities, the non-existence of second and third level health centers, the lack of a program for the timely detection of renal disease.
- There is a lack of a census of people with kidney disease in the region to improve the quality of medical care.
- People claim that the quality of services is poor: there is no equitable access, such as dialysis accessible to all sick people who demand it.
- People with kidney disease say that the disease is the main cause that increases family poverty; in order to pay for it, it is necessary to get into debt, ask for support from the community and the population in general, as well as to put aside the purchase of basic goods.
- Inhabitants are aware of the abrupt changes that the environment, especially Lake Chapala, has undergone. They understand that this circumstance has an influence on their health, their economy and their social interactions.
- The culture of the communities themselves does not allow them to talk openly about their condition, since people with kidney disease suffer from stigma from their peers. Because of this, people tend to keep quiet about their condition and avoid going to the doctor for as long as possible to avoid discrimination.
- The level of knowledge obtained allows to understand kidney disease complexity

## Data availability statement

The original contributions presented in the study are included in the article/supplementary material. Further inquiries can be directed to the corresponding author.

## Ethics statement

Ethical review and approval was not required for the study on human participants in accordance with the local legislation and institutional requirements. Written informed consent for participation was not required for this study in accordance with the national legislation and the institutional requirements.

## Author contributions

All authors listed have made a substantial, direct, and intellectual contribution to the work and approved it for publication.

## Acknowledgments

We thank each and every one of the people who voluntarily shared their time and space to tell us about their experiences with chronic kidney disease.

## Conflict of interest

The authors declare that the research was conducted in the absence of any commercial or financial relationships that could be construed as a potential conflict of interest.

## Publisher’s note

All claims expressed in this article are solely those of the authors and do not necessarily represent those of their affiliated organizations, or those of the publisher, the editors and the reviewers. Any product that may be evaluated in this article, or claim that may be made by its manufacturer, is not guaranteed or endorsed by the publisher.
